# Supplementary material for: Association between Kihon check list score and geriatric depression among older adults from ORANGE registry
Source: PLoS One. 2021 Jun 4;16(6):e0252723. doi: 10.1371/journal.pone.0252723 (PMC8177620; doi:10.1371/journal.pone.0252723)
Supplement: S1 Table — (DOCX) [file pone.0252723.s001.docx]

| **S1 Table. Items of the Kihon checklist** | | |
| --- | --- | --- |
| **No.** | **Question items** | **Score** |
| **The Kihon checklist** | |  |
| Q1: | Do you go out by bus or train by yourself? | [Yes = 0, No = 1] |
| Q2: | Do you go shopping to buy daily necessities by yourself? | [Yes = 0, No = 1] |
| Q3: | Do you manage your own deposits and savings at the bank? | [Yes = 0, No = 1] |
| Q4: | Do you sometimes visit your friends? | [Yes = 0, No = 1] |
| Q5: | Do you turn to your family or friends for advice? | [Yes = 0, No = 1] |
| Q6: | Do you normally climb stairs without using handrail or wall for support? | [Yes = 0, No = 1] |
| Q7: | Do you normally stand up from a chair without any aids? | [Yes = 0, No = 1] |
| Q8: | Do you normally walk continuously for 15 min? | [Yes = 0, No = 1] |
| Q9: | Have you experienced a fall in the past year? | [Yes = 1, No = 0] |
| Q10: | Do you have a fear of falling while walking? | [Yes = 1, No = 0] |
| Q11: | Have you lost 2 kg or more in the past 6 months? | [Yes = 1, No = 0] |
| Q12: | If BMI is less than 18.5, this item is scored. | [Yes = 1, No = 0] |
| Q13: | Do you have any difficulties eating tough foods compared to 6 months ago? | [Yes = 1, No = 0] |
| Q14: | Have you choked on your tea or soup recently? | [Yes = 1, No = 0] |
| Q15: | Do you often experience having a dry mouth? | [Yes = 1, No = 0] |
| Q16: | Do you go out at least once a week? | [Yes = 0, No = 1] |
| Q17: | Do you go out less frequently compared to last year? | [Yes = 1, No = 0] |
| Q18: | Do your family or your friends point out your memory loss? | [Yes = 1, No = 0] |
| Q19: | Do you make a call by looking up phone numbers? | [Yes = 0, No = 1] |
| Q20: | Do you find yourself not knowing today’s date? | [Yes = 1, No = 0] |
| Q21: | In the last 2 weeks have you felt a lack of fulfilment in your daily life? | [Yes = 1, No = 0] |
| Q22: | In the last 2 weeks have you felt a lack of joy when doing the things you used to enjoy? | [Yes = 1, No = 0] |
| Q23: | In the last 2 weeks have you felt difficulty in doing what you could do easily before? | [Yes = 1, No = 0] |
| Q24: | In the last 2 weeks have you felt helpless? | [Yes = 1, No = 0] |
| Q25: | In the last 2 weeks have you felt tired without a reason? | [Yes = 1, No = 0] |
| A score in the Kihon checklist (KCL) defines difficulty towards any question and a higher score of the checklist means higher risk to require support for each domain. Sub-domains of the KCL were as the follows; physical functions (a summed score of Q6 to Q10), nutritional status (Q11 and Q12), oral function (Q13 to Q15), KCL without depression score (Q1 to Q20), cognitive function (Q18 to Q20) and depressive mood (Q21 to Q25). | | |
|  |  |  |
