# Supplementary material for: Association between Kihon check list score and geriatric depression among older adults from ORANGE registry
Source: PLoS One. 2021 Jun 4;16(6):e0252723. doi: 10.1371/journal.pone.0252723 (PMC8177620; doi:10.1371/journal.pone.0252723)
Supplement: S1 Fig — (DOCX) [file pone.0252723.s002.docx]

**S1 Fig. Procedure of random intercept modeling for research subjects**

Statistical points of random intercept model to each research subject indicate as follows;

[I] Is the better GDS-15 score associated with a better KCL total score? [statistical point (SP), individual level]

[II] The lower ratio of polypharmacy or female and the faster usual walking speed (UWS) there are, does the better KCL total score become? [SP, provincial level]

[III] Is a relationship between GDS-15_CWC_ and KCL total score affected by UWS_CGM_? [SP, interaction between variables at individual level and those at provincial level]

GDS-15_CWC_

KCL score

UWS_CGM_

Cohort mean GDS-15

Provincial Level

(Level II)

Individual Level

(Level I)

[I]

[III]

[Adjustment factor]

Female dummy

Polypharmacy dummy

[II]

[II]

[II]
