# Supplementary material for: Association between Kihon check list score and geriatric depression among older adults from ORANGE registry
Source: PLoS One. 2021 Jun 4;16(6):e0252723. doi: 10.1371/journal.pone.0252723 (PMC8177620; doi:10.1371/journal.pone.0252723)
Supplement: S2 Fig — (DOCX) [file pone.0252723.s003.docx]

**S2 Fig. Coefficient (β) and significance for each variable in Model IV with a dependent variable of KCL total score**

*P < 0.05, **P < 0.01, ***P < 0.001.

The value for each item indicates coefficient (β) in Model IV with a dependent variable of KCL total score.

A result for each research subject indicates as follows;

[I] GDS-15 score at individual level was positively associated with KCL total score at individual level.

[II] The higher ratio of polypharmacy or female or the slower usual walking speed (UWS) there were, the higher KCL score became.

[III] A relationship between GDS-15_CWC_ and KCL total score at individual level was significantly affected by UWS_CGM_ at provincial level.

GDS-15_CWC_

KCL score

UWS_CGM_

[II] -2.18***

Cohort mean GDS-15

Provincial Level

(Level II)

Individual Level

(Level I)

[I] 0.55***

[III] -0.18***

[Adjustment factor] 0.55***

Female dummy

[II] 0.28 ***

Polypharmacy dummy

[II] 0.75***
