# Supplementary material for: Association between Kihon check list score and geriatric depression among older adults from ORANGE registry
Source: PLoS One. 2021 Jun 4;16(6):e0252723. doi: 10.1371/journal.pone.0252723 (PMC8177620; doi:10.1371/journal.pone.0252723)
Supplement: Attachment — Submitted filename: 2. Revised Responses to Reviwers comments for PLOS ONE (2).docx [file pone.0252723.s004.docx]

**Reviewer #1**: First of all, this study regards KCL as an evaluation of social activities, but what is the basis for this? According to the previous articles the “Kihon Checklist” (KCL), a comprehensive evaluation method, was introduced by the Japanese Ministry of Health, Labor and Welfare to identify vulnerable older adults as those at a higher risk of becoming dependent. And it has been reported this KCL is a reliable tool for predicting general frailty and frailty aspects in older adults, but it is not a tool for assessing social activities. According to these various reports the KCL is not a tool to evaluate social activity, although among the 25 items in KCL, 16 and 17 may be related the social isolation. (Geriatr Gerontol Int. 2015 Apr;15(4):518-9.; Geriatr Gerontol Int. 2016 Aug;16(8):893-902.)

**Author’s Response to reviewer’s comments;**

Thank you for supportive reviewer’s comments. Based on Satake et al. report (2016), the KCL domains reflect comprehensive functions (physical, nutritional, oral, life, cognitive and mental function) necessary for engaging in social life as shown in other references; 14. Kamegaya et al.(2017) and 15. Tomata et al.(2011). Therefore, in this study, we utilized the KCL total score as an indicator of functions necessary for engaging in social life for older adults, and called its indicator as the term of “social activity”. As pointed out by reviewer, to avoid misleading the term of “social activity”, we have described the definition related to the indicator in the Abstract (page 2, line 13) and in the section of Introduction (page 3, line 7).

**Abstract:**

“Therefore, we concluded that low walking performance and polypharmacy might play a role in poor social activities with depression in older adults.” Due to a cross-sectional study, this causal relationship cannot be determined.

**Author’s Response to reviewer’s comment of Abstract;**

Thank you for supportive reviewer’s comments. According to reviewer’s suggestion, the title and the conclusion of abstract has been revised as follows;

Abstract: “Therefore, we **suggested** that low walking performance and polypharmacy might play a role in poor social activities with depression in older adults.” (page 2, line 20)

Title: “Low walking performance and polypharmacy **are related to** poor social activities with depression in older adults” (page 1, line 2)

**Methods:**

The limitations of this manuscript are the lack of the enough information of the participants as well as sufficient descriptions in the method section.

1. Did these participants recruit from the community or on an outpatient basis?

**Author’s Response to reviewer’s comment 1 in the Methods;**

Thank you for supportive reviewer’s comments. The participants were recruited from community-dweller of five provinces in Japan. We have added the below point;

“The participants were recruited from community-dweller of five provinces in Japan.” (page 3, line 23).

2. How were the participants recruited?

**Author’s Response to reviewer’s comment 2 in the Methods;**

We have added the information regarding how the participants were recruit as the follow sentence; “The recruitments were performed by publicity papers for citizens of each province.” (page 3, line 25)

3. What is the percentage of participation in this study?

**Author’s Response to reviewer’s comment 3 in the Methods;**

Thank you for supportive reviewer’s comments. Although it is important to calculate the percentage of participant as pointed out by reviewer, we're so sorry, but the current data cannot give the participation rate in all cohorts. We would like to be able to calculate in the future.

4. How did you investigate the number of medicines in the participants? Did you use the self-reported results or did you actually count the number of drugs? Because if it is a self-report, its accuracy is questionable.

**Author’s Response to reviewer’s comment 4 in the Methods;**

We have added information about how we investigate the number of medications for each participant as the follow;

“Each participant self-reported the number of medications through the interview by medical staffs including medical doctor, public health nurses and physical or occupational therapists.” (page 4, line 17-19)

5. How was KCL implemented? Is it a self-administered or face-to-face answer?

**Author’s Response to reviewer’s comment 5 in the Methods;**

We have added the implementation of the KCL and the GDS-15 in the section of Comprehensive geriatric assessment (CGA) as follows;

“The KCL and GDS-15 were self-administered and then confirmed by face-to-face answer with the assistants for this study to prevent the missing data of each questionnaire.” (page 5, line 2-4)

6. How were grip strength and outpatient high speed measured?

**Author’s Response to reviewer’s comment 6 in the Methods;**

The assessment procedure of grip strength and usual walking speed was added in the section of Physical and cognitive measurements as follows;

“The grip strength was assessed by the hand of right and left twice, using a Smedley-type handheld dynamometer (GRIP-D; Takei Ltd., Niigata, Japan). The best value of grip strength was recorded. For measuring the UWS, participants were instructed to walk on a flat and straight surface at a comfortable walking speed, demonstrating the start and end markers of a 5-m walking distance, with a 2-m support walking distance before the start marker and after the end one. The UWS was measured twice and the fast time required was recorded.” (page 5, line 10-17)

7. How many times have these been measured?

**Author’s Response to reviewer’s comment 7 in the Methods;**

As well as “Author’s Response to reviewer’s comment 6 in the Methods”, we have added the process for measuring the physical and cognitive assessments. (page 5, line 10-17)

8. What is the cut-off value for each diagnosis of cognitive decline?

**Author’s Response to reviewer’s comment 8 in the Methods;**

The definition of cognitive impairment has been rewritten with the reference of Shimada et al. report (2016). (page 5, line 34-35, page6, line1)

**Discussion**

As can be said for all discussions, it is necessary to recognize that this study is only a cross-sectional analysis and only the relevance has been clarified, and the causal relationship is unknown.

**Author’s Response to the reviewer’s comment of discussion;**

According to reviewer’s suggestion, our conclusion was exaggerated expression. So, we have revised the expression of conclusion as suggestion. Additionally, we have changed the title and have added a sentence of limitation as the below;

Title: Low walking performance and polypharmacy are related to poor social activities with depression in older adults (page 1, line 2)

Abstract: Therefore, we suggested that low walking performance and polypharmacy might play a role in poor social activities with depression in older adults. (page 2, line 20)

Limitations: Due to a cross-sectional study, this causal relationship cannot be determined, but detailed understanding needs to be validated in a follow-up longitudinal investigation. (page 30, line 19-21)

**Reviewer #2:** The present study investigated whether social activities estimated using the Kihon checklist (KCL) score were affected by depression in older adults estimated using GDS-15. The authors further identified critical factors that affected the association of KCL score with GDS-15 by multi-level mixed-effect model analysis. As the study used multi-level mixed-effect model analysis, which provides a new perspective, it is thought that the study was conducted with sufficient knowledge of statistics, and the results are interesting, but there are some concerns about the analysis method and interpretation of the results.

1. Regarding the ORANGE registry, it is necessary to describe what kind of subjects are recruited and by what method or facility. If the registries are recruited using the same criteria, how do you explain why the five cohorts differ in various indicators?

**Author’s Response to the reviewer’s comment 1;**

Thank you for supportive reviewer’s comments. According to reviewer’s suggestion, we have described how to recruit subjects in more detail as follows;

“The recruitments were performed by publicity papers for citizens of each province.” (page 3, line 25-26)

“This study was approved by the ethics committee (approval No. 1649) of the Faculty of Medicine, Akita University and was performed in accordance with the Declaration of Helsinki II. After orally explained regarding the aim and methods of this study with the study document, all the participants were signed a consent form for this study to agree to the present study.” (page 3, line 30-34)

Regarding the question of “how do you explain why the five cohorts differ in various indicators?”, as shown in Table 1 for example, variables of the Shikoku’s cohort tend to be worse than the other cohorts in this study. The reason for this is that the study area was very depopulated. We have described the difference of depopulated rate in the section of Limitation. (page 30, line 13).

2. The authors mention in the study limitation, there were different sample sizes for each province. This means that about 77% of participants are from one of five cohorts. If the multivariate analysis is performed only on the data from the Chubu area, it will show that several factors including GDS-15 are independently related to the KCL score, but it will not be possible to find factors that explain the relationship between KCL score and GDS-15. I agree to perform a multi-level mixed-effect model analysis in this study. However, the authors need to explain in more detail in the study limitations why it is statistically meaningful to analyze provincial differences in multi-level mixed-effect model analysis and what the impact on the interpretation should be, given the population composition is so skewed toward one region.

**Author’s Response to the reviewer’s comment 2;** Thank you for supportive reviewer’s comments. The multi-level analysis is a statistical methodology represented those characteristics for each cohort, not individual features. As pointed out by reviewer, underlying its statistical method of the multi-level analysis, if only the Chubu’s cohort occupied most of the number of the target samples was analyzed using variables at the individual level, those results can’t be always necessarily similar with results in the present study. The multi-level analysis in this study was focused on revealing potential factors at provincial level, and our statistical viewpoint was different from finding of conventional multiple regression analysis. However, since the bias of results against small sample sizes of the Shikoku’s cohort in the multi-level modeling can be certain, this limitation should to be considered when interpreting our findings. Therefore, this limitation has been mentioned additionally. (Page 30, line 13-15)

3. Since the present study is a cross-sectional analysis, I don't think it is possible to know whether it is cause or effect. If a multi-level analysis is used, is it possible to determine causality? If the method of analysis could not clarify the causality, the entire description, including conclusions, should be revised to be more cautious.

**Author’s Response to the reviewer’s comment 3;**

According to reviewer’s suggestion, our conclusion was exaggerated expression. So, we have revised the expression of conclusion as suggestion. Additionally, we have changed the title and have added a sentence of limitation as the below;

Title: Low walking performance and polypharmacy are related to poor social activities with depression in older adults (page 1, line 2)

Abstract: Therefore, we suggested that low walking performance and polypharmacy might play a role in poor social activities with depression in older adults. (page 2, line 20)

Limitations: Due to a cross-sectional study, this causal relationship cannot be determined, but detailed understanding needs to be validated in a follow-up longitudinal investigation. (page 30, line 19-21)

4. KCL includes questions for depressive mood (Q21 to Q25) as you indicated in line 125. Table 2 shows that the KCL-depression score correlates with many parameters such as KCL total score (ρ=0.60), KCL-life domain (ρ=0.38), and GDS-15 (ρ=0.41). Why did the authors use GDS-15 instead of the KCL-depression score? Why did the authors investigate the effect of GDS-15 on the KCL total score which contains the depression domain in the analysis of multi-level models?

**Author’s Response to the reviewer’s comment 4;**

Thank you for supportive reviewer’s comments. As pointed out by reviewer, we used it because GDS-15 was including more detailed information on depressive status, rather than the KCL-depression score (five items of Q21 to Q25).

Also, as the reviewer pointed out, the KCL total score includes depression variables. Therefore, we confirmed the same tendency by analyzing the physical- and life-score that do not include the depression variables (Table 5,6).

5. As indicated in reference 13 by Satake, et al., the KCL is an easy yes/no self‐reporting questionnaire that has seven domains for comprehensive geriatric assessment. Each domain has a cut-off score. Satake’s study revealed that the total KCL score serves as a screening tool with which to estimate frailty, prefrailty, and robust with cut-offs of 7/8 and 3/4, but did not mention that KCL-total score can be used as a continuous quantity to assess social activities. It needs to be explained, based on previous reports or statistically, that the Spearman rank correlation coefficient can be used to quantitatively process the cumulative score of each domain, and that it is related to the degree of impairment of each domain. In particular, we can imagine that the cumulative score for each domain is correlated with the level of disability sensibly, but I wonder how we can say that the total score is correlated with social activities.

**Author’s Response to the reviewer’s comment 5;**

Based on Satake et al. report (2016), the KCL domains reflect comprehensive functions (physical, nutritional, oral, life, cognitive and mental function) necessary for engaging in social life as shown in other references; 14. Kamegaya et al.(2017) and 15. Tomata et al.(2011). Therefore, in this study, we utilized the KCL total score as an indicator of functions necessary for engaging in social life for older adults, and called its indicator as the term of “social activity”. To avoid misleading the term of “social activity”, this definition has been described in Abstract (page 2, line 13-15) and Introduction. (page 3, line 7-9)

6. Interaction between GDS-15 at individual level and provincial level variable indicates that not only interaction between GDS-15 and usual walking speed but also interaction between GDS-15 and SDST are statistically significant. The authors have not mentioned the interaction between GDS-15 and polypharmacy. How do the readers understand the meaning of interaction between GDS-15 at individual level and provincial level variable in Table 4-6.

**Author’s Response to the reviewer’s comment 6;** Thank you for supportive reviewer’s comments. The most meaningful point of multi-level modeling in this study is that variables associated with each factor of GDS-15 at individual level and GDS-15 at provincial level could be clarified. As shown in Table 5, the result of multi-level analysis could not be clarified whether two independent variables (GDS-15 and polypharmacy) which associated with KCL-physical score, were related each other or not. We are so sorry, but we would like to perform the next research project on the interaction between GDS-15 and polypharmacy in all cohorts. According to the above viewpoints, the meaning in the present study leads to why we applied GDS-15 in multi-level modeling, instead of the KCL-depression score.

7. Is there a consensus that the relevant items for the KCL-life score should be Q1-Q20? If so, please cite it in the reference. Or is it a typographical error since there is no explanation for Q16-Q17?

**Author’s Response to the reviewer’s comment 7;**

The sub-domain of Q1 to Q20 as indicated in the KCL-life score KCL has been cited in the follow references;

14. Kamegaya, T., Yamaguchi, H. and Hayashi, K. (2017). Evaluation by the Basic Checklist and the risk of 3 years incident long-term care insurance certification. J Gen Fam Med. 2017; 18, 230-236.

15. Tomata Y, Hozawa A, Ohmori-Matsuda K, et al. Validation of the Kihon Checklist for predicting the risk of 1-year incident long-term care insurance certification: the Ohsaki Cohort 2006 Study [in Japanese]. Nihon Koshu Eisei Zasshi. 2011;58:3-13.

Thus, our study applied six scores (KCL-physical score for physical functions (Q6 to Q10); KCL-nutrition score for nutritional status (Q11, 12); KCL-oral score for oral function (Q13 to Q15); KCL-life score for instrumental and social activities of daily living (Q1 to Q20); KCL-cognitive score for cognitive function (Q18 to Q20); and KCL-depressive score for depressive mood (Q21 to Q25)) of sub-domains reported by Satake et al. (2016), Kamegaya et al. (2017) and Tomata et al. (2011).

8. The Geriatric Depression Scale short-form (GDS-15) was performed by trained staff in this study (line 125-126). I understand that GDS-15 is a self-check questionnaire, should it be administered by trained staff? I think you used the Japanese version. Whose translation did you use in this study?

**Author’s Response to the reviewer’s comment 8;**

The KCL and GDS-15 were performed by face-to-face answer with well-trained assistants after self-administered to prevent the missing data of each questionnaire. The explanation of implementation has been added in the Methods. (page 5, line 2-4)

9. The citation for Paper 14 is incorrect; "Ageing Ment Health" is not needed.

**Author’s Response to the reviewer’s comment 9;**

The journal’s information of the citation for paper#14 has been revised correctly as “16. Sheikh, JI, Yesavage JA. Geriatric Depression Scale (GDS): Recent evidence and development of a shorter version. Clin Gerontol. 1986;5:165–173.” (page 33, line 8)

10. I could not find Supplementary Table 1 (line 119) in the PDF file of the submitted manuscript.

**Author’s Response to the reviewer’s comment 10;**

We have uploaded the PDF file for “Supplementary Table 1” in the resubmitted manuscript. Also, “Supplementary Table 1” has been included at the end of the resubmitted manuscript. (Page 35-36)
