# Supplementary material for: Association between Kihon check list score and geriatric depression among older adults from ORANGE registry
Source: PLoS One. 2021 Jun 4;16(6):e0252723. doi: 10.1371/journal.pone.0252723 (PMC8177620; doi:10.1371/journal.pone.0252723)
Supplement: Attachment — Submitted filename: 2. R2_Responses to Reviewers comments for PLOS ONE.docx [file pone.0252723.s005.docx]

**Reviewer #2:** I have pointed out in the previous review that there are some discrepancies between the terminology used by the authors in this paper and what is presented in the original references. The authors should reconsider the terms such as “social activity” in the conclusion of the abstract, and “the KCL-life score”. The KCL is a screening tool to assess the overall functioning of the elderly. Even if “the KCL-total score” includes indicators that affect social activity, it is not appropriate to use it as an indicator of “social activity” itself. Even though "the KCL-total score" can be an indicator of the overall functioning necessary to maintain "social activity", the more correct terminology should be used with a precise definition in the text. For example, "social capability" is not a commonly used term, and readers are likely to go back to the front of the text to find out what exactly it means. As indicated by another reviewer, the terminology of “social activity” will mislead the readers even the authors add the sentences for the definition. I strongly recommend that the authors change the terminology more correctly.

Regarding the definition or the name of “the KCL-life score” which is the sub-domain of Q1 to Q20, I could not find the word “the KCL-score” in the references that you cited in the responses. Kageyama, et al. used “the 20 Basic Checklist items” as items of Q1 to Q20 and concluded that older residents who corresponded to 10 or more of “the 20 Basic Checklist items” are at the highest risk of becoming certified as needing long-term care. The authors should reconsider the naming about "the sub-domain of Q1 to Q20", instead of “the KCL-life score”.

**Author’s Response to reviewer’s comments;**

Thank you for supportive reviewer’s comments. As pointed out by the reviewer, KCL is index containing comprehensive functions in daily life including physical, cognitive, activities of daily living (ADL) and instrumental ADL items. According to the reviewer’s recommendation, we have reconsidered the terminology in the manuscript. As pointed out by the reviewer, the terms of “social activity” and “the KCL-life score” were inadequate. Therefore, we have changed these terms to “KCL total score” itself and “KCL without depression score (Q1-20)”, according to reviewing references related to KCL as certified necessity of long-term care for older Japanese adults. We have rewritten these terms in the title, abstract, and main text.

From the cited references, I understood that “the KCL-life score” can predict prognosis with a certain cutoff value, but I could not find any data that the sum of items classified as frailty is related to prognosis as a continuous variable. I believe that using it as a continuous variable in the analysis in this paper is a new approach, but the validity and limitations of this should be described and discussed in the text.

**Author’s Response to reviewer’s comments;**

Thank you very much for supportive reviewer’s comments. As indicated by the reviewer, there is little information on a certain cutoff value of the KCL sub-domain of Q1 to Q20. Some longitudinal studies investigated predictive ability of incident long-term care insurance certification in the KCL suggested that older residents who corresponded to 10 or more of the 20 Basic Checklist items are at the highest risk of becoming certified as needing long-term care, and this variable was not continuous. According to the reviewer’s indication, we have described about this issues in the Limitations as follows;”The use of KCL score as a continuous variable in this study could be a new approach, but it has not been validated.” (Page 21, line 11-12).

Regarding the GDS-15, whose translation did you use in this study? Is It the translation by Matsubayashi or one by Sugishita? The authors should clarify it with the correct citation in the reference.

**Author’s Response to reviewer’s comments;**

Thank you for supportive reviewer’s comments. According to the reviewer’s comments, we have cited the article reported by Sugishita (2017). (Page 5, line 3 and reference 16)

**Reviewer #3:** The authors investigated the associations between the Kihon checklist (KCL) scores (total, physical and life) with individual- and provincial-level variables related to depressive symptoms, polypharmacy and cognition. The data may provide some interesting information, but the interpretation and conceptual underpinning of the analysis are questionable.

Major concerns:

1-2. I agree with previous reviewers that the KCL does not measure “social activity” in the usual sense of the phrase. The authors’ response that KCL reflects “comprehensive functions … necessary for engaging in social life” and thus is used “as an indicator of social activity” is flawed. Being alive is also necessary for engaging in social life, which, by the authors’ logic, means being alive can also be used as an indicator of social activity. Clearly no one will accept the latter interpretation, then why should one accept the former? In addition, the references provided by the authors in their response do not “show” KCL reflects the necessity for social activity; rather they use the terms frailty and insurance certification of needs for long-term care. The above point is not to say that one should not examine factors related to KCL scores. It simply says the authors should interpret the data as what the data actually represent.

**Author’s Response to reviewer’s comment 1-2;**

Thank you for supportive reviewer’s comments. As pointed out by the reviewer, the terms of “social activity” was inadequate. Therefore, we have avoided to use the term “social activity” and replaced to the term of “KCL total score” itself. We have changed these terms in the abstract and the main text.

3. More fundamentally, the authors never really defined what social activity means in the context of their study and never presented a conceptual underlying connection between provincial-level aggregated depressive symptom score, aggregated usual walking speed, and aggregated cognition (trail making test and symbol digit substitution test) with individual-level functioning (physical or life). Because of this lack of clarity, the paper is simply a collection of regression coefficients, which does not improve our understanding of the connection between these variables. For example, why is the individual-level depressive symptom positively correlated with the KCL score but, the provincial-level depressive symptom is negatively correlated with the KCL score? Also because of this lack of clarity, the authors tended to interpret the results in every which way. For example, in the discussion section, the authors first said “… these two factors (slow walking speed and polypharmacy) at the provincial and individual level affected poor social activities with depression,” and then said “the comprehensive state of social activities … has the strongest impact on individual or provincial factors.” Why should individual social activity impact provincial usual walking speed?

**Author’s Response to reviewer’s comment 3;**

Thank you very much for supportive reviewer’s comments. As pointed out by the reviewer, we are sorry that our previous interpretation of the data seems to be confused. According to the reviewer’s comments, we have re-analyzed the data using the random intercept model. Independent variables at individual- or provincial-level to put into the random intercept model were selected according to each research subject, and Supplementary Figure 2 has been added to make it easier to understand the connection between these variables. Furthermore, we have added Supplementary Figure 3 to support the interpretation toward individual variables or provincial variables. (Supplementary Figure 2 page on 28, Supplementary Figure 3 page on 29)

Statistical analysis concerns:

1. With 5 clusters, the estimation of between-cluster variation is unlikely to be accurate at all. With so few clusters, the authors attempted to estimate both the random intercept and random slope for depressive symptoms (GDS-15); thus it is not surprising the variance of the random slope could never have been truly estimated (no valid standard error) and in two of the three multi-level models the variance of the random intercept cannot be truly estimated either. In fact, with only 5 clusters, the authors used 4 cluster-level covariates in the KCL total and KCL life score models, which should exhaust the degrees of freedom at the cluster-level and make it impossible to estimate either the random intercept or the random slope; and when the authors used 3 cluster-level covariates in the KCL physical score model, they could estimate the random intercept with standard error but not random slope.

**Author’s Response to reviewer’s comment 1;**

Thank you for supportive reviewer’s comments. The current nationwide survey includes the most of cohorts in Japan except for Hokkaido, Kinki and Chugoku. Although these cohorts have different backgrounds or provincial characteristics in each nested cohort, there is little information so far on findings based on multi-level modeling method. Thus, we believe that application of the multi-level modeling can be greatly beneficial for this kind of epidemiological study. As pointed out by the reviewer, previous modeling combined with the random intercept model and the random slope model could not well documented because of too much independent variables for cluster levels. Reconsidering these issues, we have re-analyzed the random intercept modeling with an input of individual variables and provincial variables depending on each research subject as shown in Supplementary Figure 2 (page 28) .

2. The authors used the aggregated depressive symptoms, usual walking speed, trail making test and symbol digit substitution test at the provincial level as the factors of interest in Level II of the multi-level model. However, they did not control for other Level II confounding variables such as population size or population composition. In fact, with only 5 clusters, they could not control for other Level II confounders. Based on the descriptive statistics in Table 1, we can see these provinces are quite different in gender composition, which is a major confounding variable at Level II. Differences in aggregated depression, walking speed or cognition could well be due to this gender difference. I suspect if the authors had controlled for this then the other associations would be gone or significantly reduced. This is a major concern.

**Author’s Response to reviewer’s comment 2;**

Thank you for supportive reviewer’s comments. According to the reviewer’s major concerns, we have applied the random intercept model of the multilevel analysis and re-analyzed taking sex difference and adjustment of independent variables to put into the model into consideration. However, since there is a bias of higher female% for the other cohorts except for Chubu cohort as pointed out by reviewer, the limitation of the current study has been described in the section of “Limitations” (Page 21, line 7-8).

Minor concerns:

The manuscript would benefit from copyediting by a native English speaker. Some of the following items are language issues.

**Author’s Response to reviewer’s comments;**

Thank you for supportive reviewer’s comments. The manuscript has been rechecked by the native speaker (Dr. Wendy Gray from UK).

1. On page 6 line 4, “no normalization by the Kolmogorov-Smirnov test” should be that the Kolmogorov-Smirnov test was used to test the normality of variables.

**Author’s Response to reviewer’s comment 1;**

Thank you for supportive reviewer’s comments. As suggested by the reviewer, the sentence has been revised. (Page 6, line 5)

2. On page 12 Table 3, the column names should be “Estimated variance of residuals” and “Estimated variance of the random intercept.”

**Author’s Response to reviewer’s comment 2;**

Thank you for supportive reviewer’s comments. As suggested by the reviewer, the column names in Table 3 have been revised. (Page 13)

3. On page 28 line 18, the sentence should be revised to avoid confusion. For example, it can be changed to “… these two factors at the provincial- (slow walking speed) and individual-level (polypharmacy)…”

**Author’s Response to reviewer’s comment 3;**

Thank you for supportive reviewer’s comments. According to results of re-analysis using the random intercept modeling, we have simply explained about interpretation of the data with additional figures (Supplementary Figure 2 and Figure 3).

4. The phrase “poor social activities with depression” was used in multiple places, including title, abstract, and discussion. This is not accurate. Low walking speed and depressive symptom in Level II and polypharmacy in Level I are associated with KCL, but not KCL with depression.

**Author’s Response to reviewer’s comment 4;**

Thank you for supportive reviewer’s comments. As suggested by the reviewer, the phrase “poor social activities with depression” has been deleted in the manuscript. According to results of reanalysis using the random intercept modeling, all the places of title, abstract and discussion have been reconstituted accurately.

5. In the abstract, the authors stated the study was “performed from 2015 to 2019,” but in the main text it as “2013 to 2019.”

**Author’s Response to reviewer’s comment 5;**

Thank you for supportive reviewer’s comments. Our study was performed from 2013 to 2019. Thus, these sentences have been corrected. (in the abstract; page 3, line 23)

6. In table 4-6, it will be clearer to put different names for GDS-15, such as GDS-15_CGM (subscript CGM) at the provincial level and GDS-15_CWC (subscript CWC) at the individual level.

**Author’s Response to reviewer’s comment６;**

Thank you for supportive reviewer’s comments. As suggested by the reviewer, naming of GDS-15 at individual level (GDS-15-CWC) and usual walking speed (UWS) at the provincial level (UWS-CGM) has been revised in the manuscript and all the tables and figures.

7. PLOS authors have the option to publish the peer review history of their article (what does this mean?). If published, this will include your full peer review and any attached files.

**Author’s Response to reviewer’s comment 7;**

Thank you for supportive reviewer’s comments. We will have full peer review history if published.
