# Supplementary material for: Association between Kihon check list score and geriatric depression among older adults from ORANGE registry
Source: PLoS One. 2021 Jun 4;16(6):e0252723. doi: 10.1371/journal.pone.0252723 (PMC8177620; doi:10.1371/journal.pone.0252723)
Supplement: Attachment — Submitted filename: renamed_9eaff.docx [file pone.0252723.s006.docx]

**COMMENTS – Manuscript PONE-D-20-39864R2**

Title: “The association between KCL score and GDS-15 was affected by provincial factors of low walking performance, polypharmacy and sex difference”

**Important note: This review pertains only to ‘statistical aspects’ of the study and so** **‘clinical aspects’ [like medical importance, relevance of the study,** **‘clinical significance and implication(s)’ of the whole study, etc.] are to be evaluated [should be assessed] separately/independently. Further please note that any ‘statistical review’ is generally done under the assumption that (such) study specific methodological [as well as execution] issues are perfectly taken care of by the investigator(s). This review is not an exception to that and so does not cover clinical aspects {however, seldom comments are made only if those issues are intimately / scientifically related & intermingle with ‘statistical aspects’ of the study}. Agreed that ‘statistical methods’ are used as just tools here, however, they are vital part of methodology [and so should be given due importance].**

**COMMENTS:** In my opinion, your ABSTRACT is well drafted but assay type. Please note that it is preferable [refer to item 1b of CONSORT checklist 2010: Structured summary of trial design, methods, results, and conclusions] to divide the ABSTRACT with small sections like ‘Objective(s)’, ‘Methods’, ‘Results’, ‘Conclusions’, etc. which is an accepted practice of most of the good/standard journals [including this one]. It will definitely be more informative then, I guess, whatever the article type [I am aware that your Article Type: Research Article] may be.

It is well-known that the Kihon Checklist (KCL) is a self-reported comprehensive health checklist & is used as a screening tool to identify community-dwelling older adults who are vulnerable to frailty and have a higher risk of becoming dependent. Although, according to one famous systematic review ‘KCL is a reliable tool for predicting general frailty and frailty aspects in older adults (Sewo Sampaio PY, Sampaio RA, Yamada M, Arai H. Systematic review of the Kihon Checklist: Is it a reliable assessment of frailty? Geriatr Gerontol Int. 2016 Aug;16(8):893-902. doi: 10.1111/ggi.12833. PMID: 27444395), please check the ‘level of measurement’ of data yielded [KCL scores]. Please refer to lines 11-12 of page 21, ‘Limitations’ section {the use of KCL score as a continuous variable in this study could be a new approach, but it has not been validated.}.

Please note that though the measures/tools used [example: KCL score, Geriatric Depression Scale short-form (GDS-15) score, The word list memory (WM) test, trail making test version A (TMT-A) and version B (TMT-B), Symbol Digit Substitution Task (SDST), etc.] are appropriate, most of them yield data that are in [at the most] ‘ordinal’ level of measurement [and not in ratio level of measurement for sure {as the score two times higher does not indicate presence of that parameter/phenomenon as double (for example, a Visual Analogue Scales VAS score or say ‘depression’ score)}]. Then application of suitable non-parametric test(s) is/are indicated/advisable [even if distribution may be ‘Gaussian’ (i.e. normal)]. Agreed that there is/are no non-parametric test(s)/technique(s) available to be used as alternative in all situation(s) [suitable / most desired/applicable], but should be used whenever/wherever they are available. {Use of KCL score as a continuous variable in this study is surprising and calling it as ‘a new approach’ [line 11 of page 21, ‘Limitations’ section] is even more surprising.}

Therefore, use of non-parametric ‘Spearman correlation coefficient’ [in place of parametric Pearson correlation coefficient (Table 2)] is appreciated but note that ‘multi-level modelling’ is parametric (in my knowledge) which is mostly/mainly used statistical technique for this article. Are the same ‘Conclusions’ can/could be arrived at by some other technique? What is done is not wrong, however, remember that participants of your study are from the ORANGE registry (Please refer to line 2 of page 7. Why no such mention in ‘Methods – Participants? Nothing wrong in being so, but appropriate description is needed). Moreover, note that in a cross-sectional study, causal relationship cannot be determined (Please refer to line 15 of page 21, ‘Limitations’ section).

You may know (I am sure) that “Inferential statistics (i.e. hypothesis testing + estimation of CI) is built on the population model (i.e. the underlying assumption is that there is a population and we are dealing with random sample(s) drawn from that population). Although in clinical trial (involving at least two groups) we do not really deal with random samples (generally a non-probabilistic convenience sampling), ‘allocation’ to treatment groups is ‘randomly’ done which enable us to evoke the population model and we can use inferential statistics safely. But when there is only one group (so that there is no question of random allocation), with ‘non-random’ selection, it may be questionable to use inferential statistics even if you have two measurement sets as ‘pre-post’.” For a pilot study it is alright to have ‘single-arm design’, or when that is the only possibility’, however, it is very essential to keep the limitations in mind while interpreting results.

Part of ‘Conclusions’ section (lines 23-25 of page 21) that “It is possible that these factors may affect the independence of older adults not just in Japan, but in other aging countries” may be correct but is not from this study (your opinion, might have learned/realized during carrying out this study) and how can it be in ‘Conclusions’ section? Title of this article which is “**The association between KCL score and GDS-15 was affected by provincial factors of low walking performance, polypharmacy and sex difference**”, however, in my opinion, needs to be changed [especially wording, just my opinion and may be considered only if felt worthy].
