# Supplementary material for: Association between Kihon check list score and geriatric depression among older adults from ORANGE registry
Source: PLoS One. 2021 Jun 4;16(6):e0252723. doi: 10.1371/journal.pone.0252723 (PMC8177620; doi:10.1371/journal.pone.0252723)
Supplement: Attachment — Submitted filename: 2.R3_Responses to Reviewers comments for PlosOne.docx [file pone.0252723.s007.docx]

**Response to reviewer #4**

Thank you very much for supportive reviewer’s comments. According to the reviewer’s suggestion, we have revised our article as follows.

Review Comments to the Author

Reviewer #4: Important note: This review pertains only to ‘statistical aspects’ of the study and so ‘clinical aspects’ [like medical importance, relevance of the study, ‘clinical significance and implication(s)’ of the whole study, etc.] are to be evaluated [should be assessed] separately/independently. Further please note that any ‘statistical review’ is generally done under the assumption that (such) study specific methodological [as well as execution] issues are perfectly taken care of by the investigator(s). This review is not an exception to that and so does not cover clinical aspects {however, seldom comments are made only if those issues are intimately / scientifically related & intermingle with ‘statistical aspects’ of the study}. Agreed that ‘statistical methods’ are used as just tools here, however, they are vital part of methodology [and so should be given due importance].

COMMENTS: In my opinion, your ABSTRACT is well drafted but assay type. Please note that it is preferable [refer to item 1b of CONSORT checklist 2010: Structured summary of trial design, methods, results, and conclusions] to divide the ABSTRACT with small sections like ‘Objective(s)’, ‘Methods’, ‘Results’, ‘Conclusions’, etc. which is an accepted practice of most of the good/standard journals [including this one]. It will definitely be more informative then, I guess, whatever the article type [I am aware that your Article Type: Research Article] may be.

**Author’s Response to reviewer’s comments;**

Thank you very much for supportive reviewer’s comments. According to the reviewer’s suggestion, we have divided the abstract with small sections of “Objective”, “Methods”, “Results” and “Conclusions”. (Page 2, Abstract)

It is well-known that the Kihon Checklist (KCL) is a self-reported comprehensive health checklist & is used as a screening tool to identify community-dwelling older adults who are vulnerable to frailty and have a higher risk of becoming dependent. Although, according to one famous systematic review ‘KCL is a reliable tool for predicting general frailty and frailty aspects in older adults (Sewo Sampaio PY, Sampaio RA, Yamada M, Arai H. Systematic review of the Kihon Checklist: Is it a reliable assessment of frailty? Geriatr Gerontol Int. 2016 Aug;16(8):893-902. doi: 10.1111/ggi.12833. PMID: 27444395), please check the ‘level of measurement’ of data yielded [KCL scores]. Please refer to lines 11-12 of page 21, ‘Limitations’ section {the use of KCL score as a continuous variable in this study could be a new approach, but it has not been validated.}.

**Author’s Response to reviewer’s comments;**

Thank you very much for supportive reviewer’s comments. According to reviewer’s suggestion, we have added the reference (21) introduced by the reviewer and have revised the section of “Limitations” as follows; “Third, one systematic review has reported that KCL is a reliable tool for predicting general frailty and frailty aspects in older adults and there are some cut-offs for the total KCL score (Q1-Q25) to indicate general frailty (≥7 points) or to evaluate prefrail status (three out of four points) and frailty status (seven out of eight points) [28]. Compared with the total KCL score, information obtained about the KCL sub-domains are limited without satisfying validity. To evaluate the appropriateness of the KCL subdomains, the modeling divided the KCL score with sub-domains like KCL-physical score (Q6-Q10) and KCL without depression score (Q1-Q20) could be a new approach in this study.” (Page 22, line 11-19).

Please note that though the measures/tools used [example: KCL score, Geriatric Depression Scale short-form (GDS-15) score, The word list memory (WM) test, trail making test version A (TMT-A) and version B (TMT-B), Symbol Digit Substitution Task (SDST), etc.] are appropriate, most of them yield data that are in [at the most] ‘ordinal’ level of measurement [and not in ratio level of measurement for sure {as the score two times higher does not indicate presence of that parameter/phenomenon as double (for example, a Visual Analogue Scales VAS score or say ‘depression’ score)}]. Then application of suitable non-parametric test(s) is/are indicated/advisable [even if distribution may be ‘Gaussian’ (i.e. normal)]. Agreed that there is/are no non-parametric test(s)/technique(s) available to be used as alternative in all situation(s) [suitable / most desired/applicable], but should be used whenever/wherever they are available. {Use of KCL score as a continuous variable in this study is surprising and calling it as ‘a new approach’ [line 11 of page 21, ‘Limitations’ section] is even more surprising.}

**Author’s Response to reviewer’s comments;**

Thank you very much for supportive reviewer’s comments. As suggested by the reviewer, we have deleted the description of “analysis as a continuous variable” and have added the sentences of “To evaluate the appropriateness of the KCL subdomains, the modeling divided the KCL score with sub-domains like KCL-physical score (Q6-Q10) and KCL without depression score (Q1-Q20) could be a new approach in this study.” (Page 22, line 16-19) in the limitations section.

Therefore, use of non-parametric ‘Spearman correlation coefficient’ [in place of parametric Pearson correlation coefficient (Table 2)] is appreciated but note that ‘multi-level modelling’ is parametric (in my knowledge) which is mostly/mainly used statistical technique for this article. Are the same ‘Conclusions’ can/could be arrived at by some other technique? What is done is not wrong, however, remember that participants of your study are from the ORANGE registry (Please refer to line 2 of page 7. Why no such mention in ‘Methods – Participants? Nothing wrong in being so, but appropriate description is needed). Moreover, note that in a cross-sectional study, causal relationship cannot be determined (Please refer to line 15 of page 21, ‘Limitations’ section).

You may know (I am sure) that “Inferential statistics (i.e. hypothesis testing + estimation of CI) is built on the population model (i.e. the underlying assumption is that there is a population and we are dealing with random sample(s) drawn from that population). Although in clinical trial (involving at least two groups) we do not really deal with random samples (generally a non-probabilistic convenience sampling), ‘allocation’ to treatment groups is ‘randomly’ done which enable us to evoke the population model and we can use inferential statistics safely. But when there is only one group (so that there is no question of random allocation), with ‘non-random’ selection, it may be questionable to use inferential statistics even if you have two measurement sets as ‘pre-post’.” For a pilot study it is alright to have ‘single-arm design’, or when that is the only possibility’, however, it is very essential to keep the limitations in mind while interpreting results.

**Author’s Response to reviewer’s comments;**

Thank you very much for supportive reviewer’s comments. As suggested by the reviewer, the participants, we have revised the section of “Methods” including the description about “Participants” from the ORANGE registry. (Page 3, line 23-24). According to a result of the normal distribution test, the data collected from the ORANGE registry was analyzed by the non-parametric test. The above explanation has been added into the section of Methods. Based on studies previously reported by Raudenbush & Chan (1993) or Otani, Deng et al. (2020), we understand that multi-level analysis (another name; hierarchical linear model, HLM) can be applied towards the data without the normal distribution.

Also, we guess that, as commented by the reviewer, the description of “multi-level modelling’ is parametric (in my knowledge) which is mostly/mainly used statistical technique for this article” would correspond to another statistical methodology of “Hierarchical Multiple Regression Analysis：HMRA”. The HMRA is a parametric method and need to be analyzed by the data with the normal distribution. Consequently, we have applied the random intercept modeling of the HLM with the data without the normal distribution, and have added the reference 19, 20, and the explanation of “the multi-level model (hierarchical linear model, HLM)” in the section of Methods-Analyses (page 6, line 9-12).

Reference 19) Raudenbush SW, Chan WS. (1993). Application of a hierarchical linear model to the study of adolescent deviance in an overlapping cohort design. Journal of consulting and clinical psychology. 61:941-951.

Reference 20) Otani K, Deng Y, Herrmann PA, Kurz RS. (2020). Patient Satisfaction, Quality Attributes, and Organizational Characteristics: A Hierarchical Linear Model Approach. J Patient Exp. 7:801-806.

Part of ‘Conclusions’ section (lines 23-25 of page 21) that “It is possible that these factors may affect the independence of older adults not just in Japan, but in other aging countries” may be correct but is not from this study (your opinion, might have learned/realized during carrying out this study) and how can it be in ‘Conclusions’ section? Title of this article which is “The association between KCL score and GDS-15 was affected by provincial factors of low walking performance, polypharmacy and sex difference”, however, in my opinion, needs to be changed [especially wording, just my opinion and may be considered only if felt worthy].

**Author’s Response to reviewer’s comments;**

Thank you very much for supportive reviewer’s comments. Considering the reviewer’ comment, we have deleted the sentence.

Additionally, according to the reviewer’s suggestion, a title of our article has been changed revised as follows” Association between Kihon check list score and geriatric depression among older adults from Orange registry”.
